# Supplementary material for: Hot spot prediction in protein-protein interactions by an ensemble system
Source: BMC Syst Biol. 2018 Dec 31;12(Suppl 9):132. doi: 10.1186/s12918-018-0665-8 (PMC6311905; doi:10.1186/s12918-018-0665-8)
Supplement: Supplementary file 2 — All relevant MATLAB code. A simple Matlab implement of our predictor. (ZIP 19,742 kb) [file 12918_2018_665_MOESM2_ESM.zip › Additional file 2/Read ASA/ASA website/dbMPIKT/1AXIA.html]

 NetSurfP - Protein Surface Accessibility and Secondary Structure Predictions

  

|  |  |  |
| --- | --- | --- |
|  |  | NetSurfP - Protein Surface Accessibility and Secondary Structure Predictions  Technical University of Denmark |

  


---

```
# For publication of results, please cite:
# A generic method for assignment of reliability scores applied to solvent accessibility predictions.
# Bent Petersen, Thomas Nordahl Petersen, Pernille Andersen, Morten Nielsen and Claus Lundegaard
# BMC Structural Biology 2009, 9:51 doi:10.1186/1472-6807-9-51
#
# Column 1: Class assignment - B for buried or E for Exposed - Threshold: 25% exposure, but not based on RSA
# Column 2: Amino acid
# Column 3: Sequence name
# Column 4: Amino acid number
# Column 5: Relative Surface Accessibility - RSA
# Column 6: Absolute Surface Accessibility
# Column 7: Z-fit score for RSA prediction
# Column 8: Probability for Alpha-Helix
# Column 9: Probability for Beta-strand
# Column 10: Probability for Coil
E F  1AXIA                  1    0.726 145.788  -1.754   0.003   0.003   0.994
E P  1AXIA                  2    0.589  83.551  -1.029   0.018   0.019   0.964
E T  1AXIA                  3    0.606  84.066  -0.140   0.058   0.017   0.925
E I  1AXIA                  4    0.335  62.012  -0.649   0.058   0.017   0.925
E P  1AXIA                  5    0.364  51.623   0.277   0.115   0.016   0.868
B L  1AXIA                  6    0.119  21.697  -0.302   0.694   0.003   0.303
E S  1AXIA                  7    0.526  61.635   1.087   0.858   0.002   0.139
E R  1AXIA                  8    0.477 109.233   0.727   0.923   0.002   0.076
B L  1AXIA                  9    0.056  10.217   0.976   0.970   0.001   0.030
B F  1AXIA                 10    0.075  14.972   0.558   0.970   0.001   0.030
E D  1AXIA                 11    0.408  58.865   0.249   0.970   0.001   0.030
B N  1AXIA                 12    0.266  38.884   0.710   0.970   0.001   0.030
B A  1AXIA                 13    0.024   2.623   0.439   0.970   0.001   0.030
B M  1AXIA                 14    0.089  17.849  -0.176   0.970   0.001   0.030
E L  1AXIA                 15    0.309  56.578   0.317   0.970   0.001   0.030
B R  1AXIA                 16    0.118  27.022   0.611   0.970   0.001   0.030
B A  1AXIA                 17    0.024   2.645  -0.157   0.923   0.002   0.076
E H  1AXIA                 18    0.336  61.082   0.265   0.923   0.002   0.076
E R  1AXIA                 19    0.286  65.540   0.808   0.970   0.001   0.030
B L  1AXIA                 20    0.031   5.731   0.437   0.970   0.001   0.030
B H  1AXIA                 21    0.209  38.053   0.453   0.970   0.001   0.030
E Q  1AXIA                 22    0.461  82.263   1.766   0.970   0.001   0.030
B L  1AXIA                 23    0.104  18.987   1.105   0.970   0.001   0.030
B A  1AXIA                 24    0.030   3.328  -0.332   0.970   0.001   0.030
E F  1AXIA                 25    0.366  73.396   0.837   0.988   0.000   0.012
E D  1AXIA                 26    0.446  64.341   1.337   0.988   0.000   0.012
B T  1AXIA                 27    0.041   5.687   0.221   0.970   0.001   0.030
B Y  1AXIA                 28    0.119  25.516   1.049   0.970   0.001   0.030
E Q  1AXIA                 29    0.446  79.584   1.879   0.970   0.001   0.030
E E  1AXIA                 30    0.281  49.073   1.128   0.970   0.001   0.030
B F  1AXIA                 31    0.039   7.888   0.471   0.970   0.001   0.030
B E  1AXIA                 32    0.166  29.018  -0.002   0.923   0.002   0.076
E E  1AXIA                 33    0.618 108.017   0.558   0.858   0.002   0.139
E A  1AXIA                 34    0.472  52.069   0.403   0.694   0.003   0.303
B Y  1AXIA                 35    0.153  32.611   0.590   0.502   0.002   0.495
B I  1AXIA                 36    0.072  13.412  -1.377   0.181   0.016   0.803
E P  1AXIA                 37    0.330  46.799  -0.475   0.115   0.016   0.868
E K  1AXIA                 38    0.516 106.121  -1.446   0.115   0.016   0.868
E E  1AXIA                 39    0.536  93.674  -1.370   0.115   0.016   0.868
B Q  1AXIA                 40    0.253  45.186  -1.014   0.184   0.043   0.773
B K  1AXIA                 41    0.295  60.764  -0.910   0.191   0.086   0.723
B Y  1AXIA                 42    0.285  60.819  -1.125   0.307   0.165   0.527
E S  1AXIA                 43    0.307  36.016  -1.548   0.307   0.165   0.527
E F  1AXIA                 44    0.307  61.635  -1.082   0.307   0.165   0.527
B L  1AXIA                 45    0.243  44.530  -0.820   0.307   0.165   0.527
E Q  1AXIA                 46    0.367  65.528  -0.333   0.307   0.165   0.527
E N  1AXIA                 47    0.320  46.892  -1.113   0.191   0.086   0.723
E P  1AXIA                 48    0.485  68.836  -1.009   0.184   0.043   0.773
E Q  1AXIA                 49    0.349  62.260  -1.796   0.113   0.043   0.844
B T  1AXIA                 50    0.243  33.676  -1.656   0.053   0.043   0.903
E S  1AXIA                 51    0.390  45.731  -2.356   0.053   0.043   0.903
B L  1AXIA                 52    0.262  48.045  -0.816   0.052   0.084   0.864
B C  1AXIA                 53    0.066   9.238  -1.500   0.018   0.088   0.893
B F  1AXIA                 54    0.223  44.756  -0.136   0.018   0.088   0.893
E S  1AXIA                 55    0.368  43.094  -1.826   0.018   0.088   0.893
E E  1AXIA                 56    0.437  76.414  -1.539   0.019   0.141   0.840
B S  1AXIA                 57    0.270  31.667  -1.725   0.019   0.141   0.840
B I  1AXIA                 58    0.144  26.585  -0.332   0.018   0.088   0.893
E P  1AXIA                 59    0.533  75.562  -0.765   0.004   0.085   0.910
B T  1AXIA                 60    0.222  30.847  -0.306   0.005   0.015   0.979
B P  1AXIA                 61    0.239  33.957  -1.300   0.018   0.019   0.964
E S  1AXIA                 62    0.651  76.250  -0.762   0.053   0.005   0.942
E N  1AXIA                 63    0.566  82.906  -0.707   0.109   0.005   0.886
E R  1AXIA                 64    0.442 101.287  -0.409   0.406   0.004   0.590
E E  1AXIA                 65    0.629 109.816  -0.642   0.522   0.016   0.462
E E  1AXIA                 66    0.497  86.878   0.455   0.561   0.047   0.393
B T  1AXIA                 67    0.211  29.293  -0.553   0.502   0.102   0.396
E Q  1AXIA                 68    0.319  57.009   0.099   0.307   0.165   0.527
E Q  1AXIA                 69    0.661 118.072   0.331   0.199   0.152   0.649
B K  1AXIA                 70    0.161  33.035   0.666   0.191   0.086   0.723
E S  1AXIA                 71    0.548  64.272   0.010   0.053   0.043   0.903
E N  1AXIA                 72    0.391  57.301  -0.412   0.802   0.014   0.185
E L  1AXIA                 73    0.457  83.658  -0.447   0.923   0.002   0.076
E E  1AXIA                 74    0.362  63.172   0.090   0.923   0.002   0.076
B L  1AXIA                 75    0.046   8.496   0.111   0.970   0.001   0.030
B L  1AXIA                 76    0.056  10.290   0.416   0.970   0.001   0.030
E R  1AXIA                 77    0.323  73.898   0.365   0.970   0.001   0.030
B I  1AXIA                 78    0.101  18.648  -0.309   0.970   0.001   0.030
B S  1AXIA                 79    0.034   3.985  -1.064   0.970   0.001   0.030
B L  1AXIA                 80    0.117  21.386  -0.909   0.988   0.000   0.012
E L  1AXIA                 81    0.252  46.178  -0.892   0.970   0.001   0.030
B L  1AXIA                 82    0.070  12.872  -0.222   0.970   0.001   0.030
B I  1AXIA                 83    0.050   9.324  -0.535   0.923   0.002   0.076
E Q  1AXIA                 84    0.302  53.901  -0.372   0.858   0.002   0.139
B S  1AXIA                 85    0.043   4.981  -1.715   0.782   0.003   0.216
B W  1AXIA                 86    0.055  13.203  -0.451   0.600   0.003   0.397
E L  1AXIA                 87    0.255  46.599  -0.689   0.694   0.003   0.303
E E  1AXIA                 88    0.358  62.473   0.039   0.600   0.003   0.397
B P  1AXIA                 89    0.046   6.527  -0.874   0.782   0.003   0.216
B V  1AXIA                 90    0.053   8.161  -0.715   0.923   0.002   0.076
E Q  1AXIA                 91    0.386  68.957   0.812   0.923   0.002   0.076
B F  1AXIA                 92    0.215  43.171   0.548   0.970   0.001   0.030
B L  1AXIA                 93    0.024   4.376   0.881   0.923   0.002   0.076
B R  1AXIA                 94    0.194  44.380  -0.194   0.923   0.002   0.076
E S  1AXIA                 95    0.432  50.630  -0.109   0.858   0.002   0.139
B V  1AXIA                 96    0.180  27.727  -0.998   0.694   0.003   0.303
B F  1AXIA                 97    0.114  22.980  -0.869   0.406   0.004   0.590
E A  1AXIA                 98    0.434  47.849  -0.949   0.246   0.004   0.750
E N  1AXIA                 99    0.549  80.432  -2.037   0.176   0.004   0.820
E S  1AXIA                100    0.341  40.012  -2.188   0.115   0.016   0.868
E L  1AXIA                101    0.381  69.798  -1.768   0.058   0.017   0.925
E V  1AXIA                102    0.451  69.334  -1.507   0.053   0.043   0.903
B Y  1AXIA                103    0.207  44.321  -0.567   0.113   0.043   0.844
E G  1AXIA                104    0.331  26.042  -1.462   0.113   0.043   0.844
B A  1AXIA                105    0.354  38.978  -2.656   0.115   0.016   0.868
E S  1AXIA                106    0.398  46.622  -0.515   0.181   0.016   0.803
E D  1AXIA                107    0.269  38.720  -0.848   0.522   0.016   0.462
E S  1AXIA                108    0.538  63.112   0.449   0.802   0.014   0.185
E N  1AXIA                109    0.424  62.015   0.599   0.923   0.002   0.076
B V  1AXIA                110    0.043   6.640   0.083   0.970   0.001   0.030
B Y  1AXIA                111    0.236  50.390   0.311   0.970   0.001   0.030
E D  1AXIA                112    0.612  88.189   1.635   0.970   0.001   0.030
B L  1AXIA                113    0.219  40.044   1.172   0.970   0.001   0.030
B L  1AXIA                114    0.032   5.823  -0.175   0.970   0.001   0.030
E K  1AXIA                115    0.486  99.950   1.842   0.970   0.001   0.030
E D  1AXIA                116    0.420  60.565   2.026   0.970   0.001   0.030
B L  1AXIA                117    0.036   6.647   0.561   0.988   0.000   0.012
E E  1AXIA                118    0.268  46.889   0.892   0.988   0.000   0.012
E E  1AXIA                119    0.579 101.151   1.423   0.988   0.000   0.012
B R  1AXIA                120    0.168  38.358   0.429   0.988   0.000   0.012
B I  1AXIA                121    0.025   4.643   0.619   0.988   0.000   0.012
E Q  1AXIA                122    0.336  59.992   1.459   0.988   0.000   0.012
E T  1AXIA                123    0.291  40.292   1.462   0.970   0.001   0.030
B L  1AXIA                124    0.047   8.679   0.403   0.975   0.003   0.022
B M  1AXIA                125    0.045   8.944  -0.334   0.938   0.007   0.055
E G  1AXIA                126    0.456  35.887   0.805   0.879   0.010   0.111
E R  1AXIA                127    0.371  85.005   0.228   0.751   0.050   0.199
B L  1AXIA                128    0.064  11.700  -0.044   0.605   0.105   0.290
E T  1AXIA                129    0.308  42.720   0.428   0.278   0.093   0.628
E D  1AXIA                130    0.491  70.753  -0.910   0.113   0.043   0.844
E G  1AXIA                131    0.361  28.395  -1.266   0.113   0.043   0.844
B S  1AXIA                132    0.136  15.904  -1.692   0.113   0.043   0.844
E P  1AXIA                133    0.453  64.295  -1.088   0.113   0.087   0.800
E R  1AXIA                134    0.483 110.607  -1.015   0.118   0.150   0.732
E T  1AXIA                135    0.433  60.002  -1.482   0.118   0.150   0.732
E G  1AXIA                136    0.462  36.344  -2.347   0.118   0.150   0.732
E Q  1AXIA                137    0.474  84.710  -1.774   0.118   0.150   0.732
E I  1AXIA                138    0.321  59.292  -0.957   0.125   0.227   0.648
B F  1AXIA                139    0.233  46.823  -1.642   0.125   0.227   0.648
E K  1AXIA                140    0.354  72.818  -0.750   0.066   0.296   0.638
B Q  1AXIA                141    0.220  39.256  -1.134   0.066   0.296   0.638
E T  1AXIA                142    0.320  44.412  -1.832   0.064   0.216   0.721
B Y  1AXIA                143    0.177  37.910  -1.879   0.056   0.142   0.802
E S  1AXIA                144    0.308  36.156  -1.272   0.053   0.043   0.903
E K  1AXIA                145    0.552 113.505  -1.481   0.113   0.043   0.844
B F  1AXIA                146    0.206  41.384  -1.745   0.113   0.043   0.844
B D  1AXIA                147    0.304  43.850  -1.678   0.113   0.043   0.844
E T  1AXIA                148    0.418  58.018  -1.163   0.113   0.043   0.844
E N  1AXIA                149    0.344  50.332  -1.230   0.058   0.017   0.925
E S  1AXIA                150    0.444  52.084  -1.166   0.181   0.016   0.803
E H  1AXIA                151    0.645 117.253  -0.631   0.339   0.016   0.645
E N  1AXIA                152    0.503  73.683  -0.098   0.694   0.003   0.303
B D  1AXIA                153    0.222  32.005  -0.928   0.802   0.014   0.185
E D  1AXIA                154    0.406  58.548  -0.175   0.923   0.002   0.076
E A  1AXIA                155    0.412  45.369   1.060   0.923   0.002   0.076
B L  1AXIA                156    0.095  17.413   1.128   0.938   0.007   0.055
B L  1AXIA                157    0.157  28.820   0.543   0.879   0.010   0.111
E K  1AXIA                158    0.414  85.180  -0.399   0.802   0.014   0.185
B N  1AXIA                159    0.260  38.035  -0.731   0.622   0.015   0.363
B Y  1AXIA                160    0.207  44.172   0.172   0.321   0.003   0.675
B G  1AXIA                161    0.310  24.389  -1.416   0.246   0.004   0.750
B L  1AXIA                162    0.071  13.000  -0.534   0.430   0.016   0.555
B L  1AXIA                163    0.091  16.589  -0.217   0.622   0.015   0.363
B Y  1AXIA                164    0.303  64.772  -0.263   0.660   0.049   0.291
B C  1AXIA                165    0.049   6.908  -0.906   0.660   0.049   0.291
B F  1AXIA                166    0.079  15.775  -0.046   0.561   0.047   0.393
E R  1AXIA                167    0.367  84.043   0.845   0.354   0.048   0.598
E R  1AXIA                168    0.418  95.722   0.733   0.257   0.016   0.727
B D  1AXIA                169    0.158  22.825  -0.756   0.339   0.016   0.645
B M  1AXIA                170    0.107  21.451  -1.001   0.600   0.003   0.397
E T  1AXIA                171    0.476  66.021   0.126   0.858   0.002   0.139
E Y  1AXIA                172    0.241  51.395   1.706   0.923   0.002   0.076
B V  1AXIA                173    0.021   3.212   1.238   0.970   0.001   0.030
E A  1AXIA                174    0.324  35.650   0.403   0.970   0.001   0.030
B T  1AXIA                175    0.168  23.315   0.097   0.970   0.001   0.030
B Y  1AXIA                176    0.058  12.395   1.015   0.970   0.001   0.030
B L  1AXIA                177    0.057  10.345   1.089   0.975   0.003   0.022
E R  1AXIA                178    0.391  89.562   1.541   0.938   0.007   0.055
B I  1AXIA                179    0.081  14.948  -0.313   0.938   0.007   0.055
B V  1AXIA                180    0.064   9.760   0.299   0.831   0.044   0.125
E Q  1AXIA                181    0.322  57.438   0.814   0.538   0.173   0.289
B C  1AXIA                182    0.064   9.042  -0.304   0.352   0.332   0.316
E R  1AXIA                183    0.330  75.501   0.519   0.159   0.516   0.325
B S  1AXIA                184    0.242  28.362  -0.376   0.069   0.386   0.545
B V  1AXIA                185    0.204  31.386   0.546   0.064   0.216   0.721
E E  1AXIA                186    0.596 104.174   0.146   0.058   0.017   0.925
E G  1AXIA                187    0.623  49.014  -0.311   0.058   0.017   0.925
E S  1AXIA                188    0.490  57.393  -0.700   0.018   0.047   0.935
B C  1AXIA                189    0.128  17.957  -1.968   0.018   0.088   0.893
E G  1AXIA                190    0.496  39.035  -0.122   0.019   0.141   0.840
E F  1AXIA                191    0.517 103.782  -1.117   0.003   0.003   0.994

Explain the output.  Go back.
```

---
